# (Acetato-κ^2^ *O*,*O*′)bis­(1,10-phenanthroline-κ^2^ *N*,*N*′)copper(II) trifluoro­acetate tetra­hydrate

**DOI:** 10.1107/S1600536810024359

**Published:** 2010-06-30

**Authors:** Jinxia Wang, Zhuping Jin

**Affiliations:** a School of Science, North University of China, Taiyuan 030051, People’s Republic of China; b School of Chemical Engineering and Environment, North University of China, Taiyuan 030051, People’s Republic of China

## Abstract

In the title compound, [Cu(CH_3_CO_2_)(C_12_H_8_N_2_)_2_](CF_3_CO_2_)·4H_2_O, the Cu^II^ atom shows a distorted octa­hedral coordination with four N atoms [Cu—N = 2.015 (3)–2.244 (3) Å] from the two phenanthroline ligands and two O atoms from the acetate [Cu—O = 1.953 (3) and 2.764 (3) Å]. Strong inter­molecular O—H⋯O hydrogen-bonding inter­actions consolidate the crystal packing. The F atoms of the anion are disordered over two positions in a 0.5233 (3):0.4767 (3) ratio.

## Related literature

For metal–1,10-phenanthroline complexes with carboxyl­ates, see: Sun *et al.* (2007 ▶); Liu *et al.* (2009 ▶).
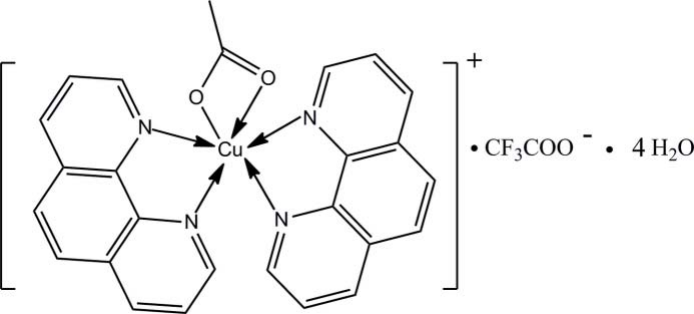


## Experimental

### 

#### Crystal data

[Cu(C_2_H_3_O_2_)(C_12_H_8_N_2_)_2_](C_2_F_3_O_2_)·4H_2_O
*M* *_r_* = 668.08
Triclinic, 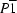

*a* = 8.9019 (7) Å
*b* = 11.6662 (9) Å
*c* = 15.698 (1) Å
α = 101.619 (1)°
β = 101.512 (1)°
γ = 108.514 (1)°
*V* = 1451.98 (19) Å^3^
*Z* = 2
Mo *K*α radiation
μ = 0.83 mm^−1^
*T* = 293 K
0.28 × 0.25 × 0.19 mm

#### Data collection

Bruker SMART APEX diffractometer
Absorption correction: multi-scan (*SADABS*; Bruker, 2005 ▶) *T*_min_ = 0.801, *T*_max_ = 0.859
7689 measured reflections
5112 independent reflections
4389 reflections with *I* > 2σ(*I*)
*R*_int_ = 0.043

#### Refinement

*R*[*F* ^2^ > 2σ(*F* ^2^)] = 0.051
*wR*(*F* ^2^) = 0.159
*S* = 1.02
5112 reflections
425 parameters
103 restraints
H-atom parameters constrained
Δρ_max_ = 1.04 e Å^−3^
Δρ_min_ = −0.87 e Å^−3^

### 

Data collection: *SMART* (Bruker, 2005 ▶); cell refinement: *SAINT* (Bruker, 2005 ▶); data reduction: *SAINT*; program(s) used to solve structure: *SHELXS97* (Sheldrick, 2008 ▶); program(s) used to refine structure: *SHELXL97* (Sheldrick, 2008 ▶); molecular graphics: *XP* in *SHELXTL* (Sheldrick, 2008 ▶); software used to prepare material for publication: *SHELXL97*.

## Supplementary Material

Crystal structure: contains datablocks I, global. DOI: 10.1107/S1600536810024359/ez2212sup1.cif

Structure factors: contains datablocks I. DOI: 10.1107/S1600536810024359/ez2212Isup2.hkl

Additional supplementary materials:  crystallographic information; 3D view; checkCIF report

## Figures and Tables

**Table:** 

Cu1—O1	1.953 (3)
Cu1—N1	2.015 (3)
Cu1—N4	2.022 (3)
Cu1—N2	2.037 (3)
Cu1—N3	2.244 (3)

**Table:** 

O1—Cu1—N1	91.55 (13)
O1—Cu1—N4	93.66 (13)
N1—Cu1—N4	169.21 (13)
O1—Cu1—N2	171.51 (12)
N1—Cu1—N2	81.21 (14)
N4—Cu1—N2	92.78 (14)
O1—Cu1—N3	93.94 (12)
N1—Cu1—N3	110.64 (13)
N4—Cu1—N3	78.44 (13)
N2—Cu1—N3	92.75 (13)

**Table 2:** Hydrogen-bond geometry (Å, °)

*D*—H⋯*A*	*D*—H	H⋯*A*	*D*⋯*A*	*D*—H⋯*A*
O7—H7*A*⋯O5	0.85	1.95	2.787 (8)	168
O7—H7*B*⋯O4	0.85	2.11	2.854 (8)	145
O6—H6*A*⋯O1	0.85	2.00	2.844 (5)	170
O6—H6*B*⋯O4^i^	0.85	2.03	2.881 (7)	175
O8—H8*B*⋯O3^ii^	0.85	2.03	2.868 (10)	171
O8—H8*A*⋯O6^iii^	0.85	2.17	2.809 (9)	132

Symmetry codes: (i) 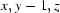; (ii) 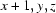; (iii) 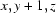.

**Fig. 1.**
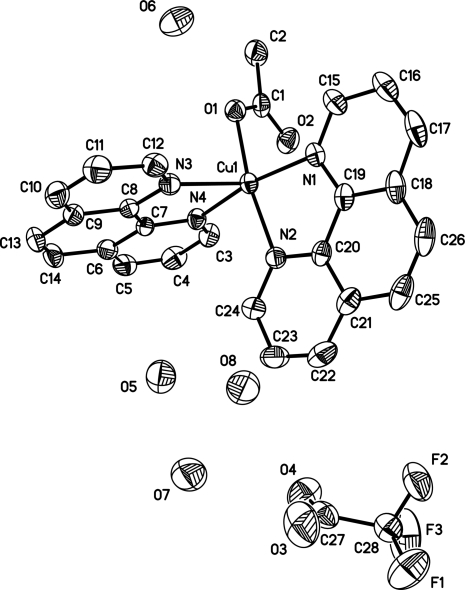
The molecular structure of title compound, with atom labels and 30% probability displacement ellipsoids for non-H atoms.

## supplementary crystallographic information

### Comment

Metal complexes with carboxylates are among the most investigated complexes in the field of coordination chemistry, in which metal-1,10-phenanthroline complexes and their derivatives have also attracted much attention during recent decades because of their interesting features (Sun *et al.*, 2007; Liu *et al.*, 2009). In this work, the title compound was obtained by the reaction of trifluoroacetic acid and cupric acetate in the presence of 1,10-phenanthroline as co-ligand.

The molecular structure of the title complex is shown in Fig. 1. The Cu^II^ atom exhibits a six-coordinate distorted octahedral geometry with four N atoms [Cu—N 2.015 (3) Å–2.244 (3) Å] from two phenanthroline ligands and two O atoms from the acetate ligand [Cu—O 1.953 (3), 2.764 (3) Å]. Three N atoms and one O atom occupy the equatorial positions with a slight departure from the ideal plane by 0.0563 (2) Å, while one O atom and one N atom lie in the apical positions with an axis angle of 140.63 (10)°, showing a large deviation from the expected 180°. Strong intermolecular O—H···O hydrogen bonding interactions exist.

### Experimental

The reaction was carried out by the solvothermal method. Trifluoroacetic acid (0.114 g,1 mmol) and cupric acetate (0.199 g, 1 mmol) and 1,10-phenanthroline (0.312 g, 2 mmol) were added to an airtight vessel with the 21 ml of a 2:1 ethanol-water mixture. The resulting blue solution was filtered. Upon standing, the filtrate yielded blue block-shaped crystals after several days.

The yield is 76% and elemental analysis: calc. for C_28_H_27_CuF_3_N_4_O_8_: C 50.34, H 4.07, N 8.39; found: C 50.52, H 4.29, N 8.53. The elemental analyses were performed with PERKIN ELMER MODEL 2400 SERIES II.

### Refinement

The *U*_iso_(H) values were set at 1.2*U*_eq_(C—H) for the H atoms from the phen rings and waters, 1.5*U*_eq_(C—H) for the methyl moiety. As the diffraction intensities were of high quality, the H atoms could be located in difference Fourier maps and refined using the riding model. Three disordered F atoms were treated as statistically disordered between two positions with the refined occupancies of 0.5233 (3) and 0.4767 (3), respectively.

### Crystal data

**Table:**

[Cu(C_2_H_3_O_2_)(C_12_H_8_N_2_)_2_](C_2_F_3_O_2_)·4H_2_O	*Z* = 2
*M**_r_* = 668.08	*F*(000) = 686
Triclinic, *P*1	*D*_x_ = 1.528 Mg m^−^^3^
*a* = 8.9019 (7) Å	Mo *K*α radiation, λ = 0.71073 Å
*b* = 11.6662 (9) Å	Cell parameters from 4430 reflections
*c* = 15.698 (1) Å	θ = 2.5–27.8°
α = 101.619 (1)°	µ = 0.83 mm^−^^1^
β = 101.512 (1)°	*T* = 293 K
γ = 108.514 (1)°	Block, blue
*V* = 1451.98 (19) Å^3^	0.28 × 0.25 × 0.19 mm

### Data collection

**Table:**

Bruker SMART APEX diffractometer	5112 independent reflections
Radiation source: fine-focus sealed tube	4389 reflections with *I* > 2σ(*I*)
graphite	*R*_int_ = 0.043
phi and ω scans	θ_max_ = 25.1°, θ_min_ = 2.5°
Absorption correction: multi-scan (*SADABS*; Bruker, 2005)	*h* = −10→10
*T*_min_ = 0.801, *T*_max_ = 0.859	*k* = −13→13
7689 measured reflections	*l* = −18→17

### Refinement

**Table:**

Refinement on *F*^2^	Primary atom site location: structure-invariant direct methods
Least-squares matrix: full	Secondary atom site location: difference Fourier map
*R*[*F*^2^ > 2σ(*F*^2^)] = 0.051	Hydrogen site location: inferred from neighbouring sites
*wR*(*F*^2^) = 0.159	H-atom parameters constrained
*S* = 1.02	*w* = 1/[σ^2^(*F*_o_^2^) + (0.108*P*)^2^ + 0.7243*P*] where *P* = (*F*_o_^2^ + 2*F*_c_^2^)/3
5112 reflections	(Δ/σ)_max_ = 0.002
425 parameters	Δρ_max_ = 1.04 e Å^−^^3^
103 restraints	Δρ_min_ = −0.86 e Å^−^^3^

### Special details

**Table:**

Geometry. All e.s.d.'s (except the e.s.d. in the dihedral angle between two l.s. planes) are estimated using the full covariance matrix. The cell e.s.d.'s are taken into account individually in the estimation of e.s.d.'s in distances, angles and torsion angles; correlations between e.s.d.'s in cell parameters are only used when they are defined by crystal symmetry. An approximate (isotropic) treatment of cell e.s.d.'s is used for estimating e.s.d.'s involving l.s. planes.
Refinement. Refinement of *F*^2^ against ALL reflections. The weighted *R*-factor *wR* and goodness of fit *S* are based on *F*^2^, conventional *R*-factors *R* are based on *F*, with *F* set to zero for negative *F*^2^. The threshold expression of *F*^2^ > σ(*F*^2^) is used only for calculating *R*-factors(gt) *etc*. and is not relevant to the choice of reflections for refinement. *R*-factors based on *F*^2^ are statistically about twice as large as those based on *F*, and *R*- factors based on ALL data will be even larger.

### Fractional atomic coordinates and isotropic or equivalent isotropic displacement parameters (Å^2^)

**Table:**

	*x*	*y*	*z*	*U*_iso_*/*U*_eq_	Occ. (<1)
Cu1	0.70769 (5)	0.42710 (4)	0.71713 (3)	0.0397 (2)	
F1	0.083 (2)	0.9968 (17)	0.6171 (10)	0.133 (4)	0.52 (2)
F2	0.138 (2)	0.8377 (11)	0.5566 (10)	0.118 (4)	0.52 (2)
F3	0.3252 (15)	1.0243 (15)	0.6021 (9)	0.121 (4)	0.52 (2)
F1'	0.0317 (13)	0.9385 (17)	0.5837 (12)	0.120 (4)	0.48 (2)
F2'	0.206 (2)	0.8680 (17)	0.5546 (10)	0.117 (4)	0.48 (2)
F3'	0.263 (2)	1.0720 (11)	0.6148 (9)	0.116 (4)	0.48 (2)
N1	0.5545 (4)	0.3202 (3)	0.5937 (2)	0.0432 (8)	
N2	0.5668 (4)	0.5324 (3)	0.6982 (2)	0.0440 (8)	
N3	0.6031 (4)	0.3667 (3)	0.8268 (2)	0.0439 (8)	
N4	0.8712 (4)	0.5606 (3)	0.8296 (2)	0.0428 (8)	
O1	0.8368 (4)	0.3195 (3)	0.7158 (2)	0.0491 (7)	
O2	0.9846 (4)	0.4555 (4)	0.6563 (2)	0.0676 (9)	
O3	0.1324 (9)	0.8811 (7)	0.7380 (5)	0.146 (2)	
O4	0.3983 (7)	0.9689 (5)	0.7462 (4)	0.1105 (16)	
O5	0.7803 (6)	0.8614 (5)	0.9518 (4)	0.1055 (15)	
H5A	0.8130	0.8679	0.9051	0.127*	
H5B	0.8480	0.9207	0.9978	0.127*	
O6	0.7421 (6)	0.0847 (4)	0.7568 (4)	0.0954 (14)	
H6A	0.7587	0.1508	0.7392	0.115*	
H6B	0.6419	0.0542	0.7570	0.115*	
O7	0.5361 (8)	0.9627 (7)	0.9249 (4)	0.139 (2)	
H7A	0.6116	0.9327	0.9256	0.166*	
H7B	0.5276	0.9963	0.8818	0.166*	
O8	0.9949 (10)	1.0217 (8)	0.8494 (5)	0.176 (3)	
H8A	0.8909	0.9997	0.8294	0.211*	
H8B	1.0276	0.9799	0.8113	0.211*	
C1	0.9569 (5)	0.3597 (5)	0.6812 (3)	0.0505 (10)	
C2	1.0606 (7)	0.2817 (6)	0.6725 (4)	0.0720 (15)	
H2A	1.0192	0.2109	0.6951	0.108*	
H2B	1.1731	0.3315	0.7071	0.108*	
H2C	1.0556	0.2522	0.6099	0.108*	
C3	1.0023 (6)	0.6569 (4)	0.8305 (3)	0.0539 (11)	
H3	1.0226	0.6644	0.7757	0.065*	
C4	1.1096 (6)	0.7466 (4)	0.9102 (4)	0.0624 (13)	
H4	1.1998	0.8126	0.9082	0.075*	
C5	1.0821 (6)	0.7371 (4)	0.9907 (3)	0.0583 (12)	
H5	1.1533	0.7966	1.0444	0.070*	
C6	0.9449 (5)	0.6366 (4)	0.9928 (3)	0.0497 (10)	
C7	0.8417 (5)	0.5507 (4)	0.9101 (3)	0.0402 (9)	
C8	0.6997 (5)	0.4478 (4)	0.9087 (3)	0.0401 (9)	
C9	0.6667 (6)	0.4339 (4)	0.9899 (3)	0.0511 (11)	
C10	0.5254 (7)	0.3300 (5)	0.9842 (4)	0.0659 (14)	
H10	0.4985	0.3174	1.0368	0.079*	
C11	0.4303 (7)	0.2496 (5)	0.9031 (4)	0.0676 (14)	
H11	0.3378	0.1809	0.8992	0.081*	
C12	0.4720 (6)	0.2712 (4)	0.8253 (3)	0.0544 (11)	
H12	0.4047	0.2153	0.7694	0.065*	
C13	0.7751 (7)	0.5228 (5)	1.0731 (3)	0.0633 (13)	
H13	0.7536	0.5136	1.1274	0.076*	
C14	0.9071 (7)	0.6189 (5)	1.0746 (3)	0.0627 (13)	
H14	0.9755	0.6753	1.1301	0.075*	
C15	0.5530 (6)	0.2158 (4)	0.5418 (3)	0.0566 (11)	
H15	0.6303	0.1826	0.5631	0.068*	
C16	0.4407 (7)	0.1531 (5)	0.4566 (3)	0.0685 (14)	
H16	0.4453	0.0806	0.4214	0.082*	
C17	0.3246 (7)	0.1982 (6)	0.4250 (3)	0.0719 (16)	
H17	0.2476	0.1556	0.3687	0.086*	
C18	0.3210 (5)	0.3089 (5)	0.4771 (3)	0.0568 (12)	
C19	0.4408 (5)	0.3674 (4)	0.5617 (3)	0.0446 (9)	
C20	0.4465 (5)	0.4808 (4)	0.6192 (3)	0.0435 (9)	
C21	0.3284 (5)	0.5328 (5)	0.5919 (3)	0.0549 (11)	
C22	0.3376 (7)	0.6413 (6)	0.6535 (4)	0.0709 (15)	
H22	0.2617	0.6788	0.6392	0.085*	
C23	0.4577 (8)	0.6925 (5)	0.7343 (4)	0.0720 (15)	
H23	0.4622	0.7637	0.7757	0.086*	
C24	0.5729 (6)	0.6375 (5)	0.7545 (3)	0.0585 (12)	
H24	0.6565	0.6751	0.8090	0.070*	
C25	0.2096 (6)	0.4726 (6)	0.5058 (4)	0.0689 (16)	
H25	0.1326	0.5073	0.4870	0.083*	
C26	0.2068 (6)	0.3658 (7)	0.4505 (4)	0.0698 (16)	
H26	0.1285	0.3295	0.3941	0.084*	
C27	0.2466 (9)	0.9333 (5)	0.7120 (4)	0.0738 (16)	
C28	0.1951 (7)	0.9529 (5)	0.6193 (4)	0.0709 (14)	

### Atomic displacement parameters (Å^2^)

**Table:**

	*U*^11^	*U*^22^	*U*^33^	*U*^12^	*U*^13^	*U*^23^
Cu1	0.0400 (3)	0.0424 (3)	0.0318 (3)	0.0131 (2)	0.0061 (2)	0.0079 (2)
F1	0.127 (8)	0.135 (8)	0.138 (8)	0.076 (6)	−0.004 (6)	0.033 (6)
F2	0.108 (8)	0.122 (6)	0.075 (5)	−0.001 (6)	0.009 (6)	0.010 (5)
F3	0.109 (7)	0.130 (7)	0.119 (6)	0.005 (6)	0.049 (5)	0.070 (6)
F1'	0.071 (6)	0.127 (8)	0.130 (8)	0.013 (6)	0.004 (5)	0.032 (6)
F2'	0.118 (8)	0.148 (8)	0.071 (5)	0.060 (7)	0.024 (6)	−0.015 (6)
F3'	0.127 (8)	0.102 (7)	0.094 (6)	0.006 (6)	0.018 (6)	0.050 (5)
N1	0.0406 (18)	0.0477 (19)	0.0347 (17)	0.0091 (15)	0.0117 (14)	0.0089 (15)
N2	0.0454 (19)	0.0455 (19)	0.0408 (18)	0.0165 (15)	0.0121 (15)	0.0125 (15)
N3	0.0445 (19)	0.0401 (18)	0.0436 (19)	0.0126 (15)	0.0115 (15)	0.0108 (15)
N4	0.0416 (18)	0.0445 (18)	0.0366 (17)	0.0123 (15)	0.0057 (14)	0.0110 (15)
O1	0.0505 (17)	0.0550 (17)	0.0462 (16)	0.0231 (14)	0.0162 (13)	0.0159 (14)
O2	0.072 (2)	0.080 (2)	0.066 (2)	0.0317 (19)	0.0306 (18)	0.038 (2)
O3	0.175 (6)	0.155 (6)	0.132 (5)	0.042 (5)	0.091 (5)	0.077 (5)
O4	0.098 (4)	0.090 (3)	0.113 (4)	0.023 (3)	−0.004 (3)	0.020 (3)
O5	0.093 (3)	0.131 (4)	0.099 (3)	0.045 (3)	0.031 (3)	0.038 (3)
O6	0.090 (3)	0.075 (3)	0.127 (4)	0.027 (2)	0.034 (3)	0.044 (3)
O7	0.136 (5)	0.190 (6)	0.109 (4)	0.102 (5)	0.023 (4)	0.029 (4)
O8	0.203 (8)	0.259 (9)	0.161 (7)	0.159 (7)	0.096 (6)	0.099 (7)
C1	0.051 (2)	0.068 (3)	0.034 (2)	0.025 (2)	0.0098 (18)	0.016 (2)
C2	0.076 (3)	0.102 (4)	0.062 (3)	0.053 (3)	0.032 (3)	0.030 (3)
C3	0.051 (3)	0.051 (2)	0.051 (3)	0.009 (2)	0.008 (2)	0.018 (2)
C4	0.047 (3)	0.045 (3)	0.074 (3)	0.004 (2)	0.001 (2)	0.012 (2)
C5	0.053 (3)	0.048 (3)	0.052 (3)	0.015 (2)	−0.007 (2)	−0.003 (2)
C6	0.052 (2)	0.052 (2)	0.042 (2)	0.026 (2)	0.0027 (19)	0.0063 (19)
C7	0.044 (2)	0.043 (2)	0.0327 (19)	0.0205 (18)	0.0040 (16)	0.0087 (16)
C8	0.045 (2)	0.044 (2)	0.038 (2)	0.0233 (18)	0.0126 (17)	0.0132 (17)
C9	0.062 (3)	0.062 (3)	0.046 (2)	0.036 (2)	0.023 (2)	0.021 (2)
C10	0.075 (3)	0.078 (4)	0.068 (3)	0.035 (3)	0.041 (3)	0.037 (3)
C11	0.066 (3)	0.060 (3)	0.083 (4)	0.019 (3)	0.036 (3)	0.029 (3)
C12	0.054 (3)	0.044 (2)	0.061 (3)	0.012 (2)	0.020 (2)	0.014 (2)
C13	0.086 (4)	0.079 (4)	0.036 (2)	0.043 (3)	0.022 (2)	0.017 (2)
C14	0.075 (3)	0.078 (3)	0.033 (2)	0.039 (3)	0.005 (2)	0.004 (2)
C15	0.058 (3)	0.053 (3)	0.048 (3)	0.010 (2)	0.018 (2)	0.004 (2)
C16	0.070 (3)	0.063 (3)	0.049 (3)	0.005 (3)	0.020 (2)	−0.003 (2)
C17	0.056 (3)	0.086 (4)	0.034 (2)	−0.008 (3)	0.005 (2)	−0.002 (2)
C18	0.041 (2)	0.078 (3)	0.037 (2)	0.001 (2)	0.0096 (18)	0.019 (2)
C19	0.034 (2)	0.056 (2)	0.038 (2)	0.0049 (18)	0.0116 (16)	0.0184 (19)
C20	0.036 (2)	0.055 (2)	0.042 (2)	0.0124 (18)	0.0152 (17)	0.0231 (19)
C21	0.043 (2)	0.071 (3)	0.064 (3)	0.022 (2)	0.022 (2)	0.039 (3)
C22	0.066 (3)	0.079 (4)	0.096 (4)	0.043 (3)	0.035 (3)	0.050 (3)
C23	0.086 (4)	0.062 (3)	0.083 (4)	0.041 (3)	0.032 (3)	0.022 (3)
C24	0.068 (3)	0.056 (3)	0.055 (3)	0.029 (2)	0.017 (2)	0.013 (2)
C25	0.039 (2)	0.108 (5)	0.071 (3)	0.024 (3)	0.015 (2)	0.056 (4)
C26	0.037 (2)	0.115 (5)	0.047 (3)	0.010 (3)	0.005 (2)	0.038 (3)
C27	0.094 (4)	0.050 (3)	0.073 (4)	0.018 (3)	0.036 (3)	0.010 (3)
C28	0.069 (3)	0.065 (3)	0.074 (4)	0.015 (3)	0.028 (3)	0.020 (3)

### Geometric parameters (Å, °)

**Table:**

Cu1—O1	1.953 (3)	C4—H4	0.9300
Cu1—N1	2.015 (3)	C5—C6	1.410 (7)
Cu1—N4	2.022 (3)	C5—H5	0.9300
Cu1—N2	2.037 (3)	C6—C7	1.397 (6)
Cu1—N3	2.244 (3)	C6—C14	1.428 (7)
F1—C28	1.256 (11)	C7—C8	1.433 (6)
F2—C28	1.367 (12)	C8—C9	1.392 (6)
F3—C28	1.308 (10)	C9—C10	1.415 (7)
F1'—C28	1.393 (12)	C9—C13	1.425 (7)
F2'—C28	1.311 (12)	C10—C11	1.343 (8)
F3'—C28	1.351 (11)	C10—H10	0.9301
N1—C15	1.317 (6)	C11—C12	1.392 (7)
N1—C19	1.357 (5)	C11—H11	0.9300
N2—C24	1.337 (6)	C12—H12	0.9300
N2—C20	1.344 (5)	C13—C14	1.334 (8)
N3—C12	1.326 (6)	C13—H13	0.9300
N3—C8	1.357 (5)	C14—H14	0.9300
N4—C3	1.333 (6)	C15—C16	1.389 (7)
N4—C7	1.361 (5)	C15—H15	0.9300
O1—C1	1.294 (5)	C16—C17	1.355 (8)
O2—C1	1.226 (6)	C16—H16	0.9300
O3—C27	1.202 (8)	C17—C18	1.396 (8)
O4—C27	1.245 (8)	C17—H17	0.9300
O5—H5A	0.8500	C18—C19	1.406 (6)
O5—H5B	0.8500	C18—C26	1.422 (8)
O6—H6A	0.8501	C19—C20	1.423 (6)
O6—H6B	0.8500	C20—C21	1.410 (6)
O7—H7A	0.8500	C21—C22	1.397 (8)
O7—H7B	0.8501	C21—C25	1.419 (7)
O8—H8A	0.8500	C22—C23	1.363 (8)
O8—H8B	0.8500	C22—H22	0.9300
C1—C2	1.494 (7)	C23—C24	1.391 (7)
C2—H2A	0.9600	C23—H23	0.9300
C2—H2B	0.9600	C24—H24	0.9300
C2—H2C	0.9600	C25—C26	1.356 (8)
C3—C4	1.390 (7)	C25—H25	0.9300
C3—H3	0.9300	C26—H26	0.9300
C4—C5	1.353 (8)	C27—C28	1.521 (9)
			
O1—Cu1—N1	91.55 (13)	C14—C13—C9	121.3 (4)
O1—Cu1—N4	93.66 (13)	C14—C13—H13	119.3
N1—Cu1—N4	169.21 (13)	C9—C13—H13	119.4
O1—Cu1—N2	171.51 (12)	C13—C14—C6	121.3 (4)
N1—Cu1—N2	81.21 (14)	C13—C14—H14	119.2
N4—Cu1—N2	92.78 (14)	C6—C14—H14	119.6
O1—Cu1—N3	93.94 (12)	N1—C15—C16	122.8 (5)
N1—Cu1—N3	110.64 (13)	N1—C15—H15	118.5
N4—Cu1—N3	78.44 (13)	C16—C15—H15	118.7
N2—Cu1—N3	92.75 (13)	C17—C16—C15	119.6 (5)
C15—N1—C19	117.9 (4)	C17—C16—H16	120.2
C15—N1—Cu1	129.2 (3)	C15—C16—H16	120.2
C19—N1—Cu1	112.9 (3)	C16—C17—C18	120.0 (4)
C24—N2—C20	118.2 (4)	C16—C17—H17	120.1
C24—N2—Cu1	129.2 (3)	C18—C17—H17	120.0
C20—N2—Cu1	112.5 (3)	C17—C18—C19	116.7 (5)
C12—N3—C8	117.7 (4)	C17—C18—C26	124.9 (5)
C12—N3—Cu1	132.8 (3)	C19—C18—C26	118.4 (5)
C8—N3—Cu1	109.5 (2)	N1—C19—C18	123.0 (4)
C3—N4—C7	118.3 (4)	N1—C19—C20	116.5 (4)
C3—N4—Cu1	125.3 (3)	C18—C19—C20	120.5 (4)
C7—N4—Cu1	116.4 (3)	N2—C20—C21	123.5 (4)
C1—O1—Cu1	110.9 (3)	N2—C20—C19	116.9 (4)
H5A—O5—H5B	109.6	C21—C20—C19	119.6 (4)
H6A—O6—H6B	109.6	C22—C21—C20	116.4 (4)
H7A—O7—H7B	109.8	C22—C21—C25	124.8 (5)
H8A—O8—H8B	108.6	C20—C21—C25	118.8 (5)
O2—C1—O1	122.5 (4)	C23—C22—C21	120.3 (5)
O2—C1—C2	121.4 (4)	C23—C22—H22	119.8
O1—C1—C2	116.0 (4)	C21—C22—H22	119.9
C1—C2—H2A	109.0	C22—C23—C24	119.6 (5)
C1—C2—H2B	109.7	C22—C23—H23	120.1
H2A—C2—H2B	109.5	C24—C23—H23	120.3
C1—C2—H2C	109.8	N2—C24—C23	122.0 (5)
H2A—C2—H2C	109.5	N2—C24—H24	119.3
H2B—C2—H2C	109.5	C23—C24—H24	118.7
N4—C3—C4	122.7 (4)	C26—C25—C21	121.3 (5)
N4—C3—H3	118.7	C26—C25—H25	119.6
C4—C3—H3	118.7	C21—C25—H25	119.2
C5—C4—C3	119.5 (5)	C25—C26—C18	121.4 (5)
C5—C4—H4	120.3	C25—C26—H26	119.1
C3—C4—H4	120.2	C18—C26—H26	119.5
C4—C5—C6	119.7 (4)	O3—C27—O4	130.9 (7)
C4—C5—H5	120.2	O3—C27—C28	113.8 (7)
C6—C5—H5	120.1	O4—C27—C28	115.2 (6)
C7—C6—C5	117.6 (4)	F1—C28—F3	113.6 (9)
C7—C6—C14	118.9 (4)	F1—C28—F2'	127.6 (11)
C5—C6—C14	123.5 (4)	F3—C28—F2'	81.4 (9)
N4—C7—C6	122.2 (4)	F1—C28—F3'	74.3 (9)
N4—C7—C8	118.1 (3)	F3—C28—F3'	40.5 (6)
C6—C7—C8	119.6 (4)	F2'—C28—F3'	113.5 (10)
N3—C8—C9	122.7 (4)	F1—C28—F2	110.5 (9)
N3—C8—C7	117.6 (3)	F3—C28—F2	106.9 (8)
C9—C8—C7	119.7 (4)	F2'—C28—F2	26.1 (8)
C8—C9—C10	117.2 (4)	F3'—C28—F2	132.9 (10)
C8—C9—C13	119.1 (4)	F1—C28—F1'	30.7 (7)
C10—C9—C13	123.7 (4)	F3—C28—F1'	126.8 (10)
C11—C10—C9	120.0 (5)	F2'—C28—F1'	99.2 (9)
C11—C10—H10	120.0	F3'—C28—F1'	96.1 (9)
C9—C10—H10	120.0	F2—C28—F1'	79.8 (9)
C10—C11—C12	119.0 (5)	F1—C28—C27	109.3 (8)
C10—C11—H11	120.3	F3—C28—C27	109.3 (8)
C12—C11—H11	120.7	F2'—C28—C27	112.0 (9)
N3—C12—C11	123.4 (5)	F3'—C28—C27	115.4 (7)
N3—C12—H12	118.3	F2—C28—C27	107.1 (8)
C11—C12—H12	118.3	F1'—C28—C27	119.0 (8)

### Hydrogen-bond geometry (Å, °)

**Table:**

*D*—H···*A*	*D*—H	H···*A*	*D*···*A*	*D*—H···*A*
O7—H7A···O5	0.85	1.95	2.787 (8)	168
O7—H7B···O4	0.85	2.11	2.854 (8)	145
O6—H6A···O1	0.85	2.00	2.844 (5)	170
O6—H6B···O4^i^	0.85	2.03	2.881 (7)	175
O8—H8B···O3^ii^	0.85	2.03	2.868 (10)	171
O8—H8A···O6^iii^	0.85	2.17	2.809 (9)	132

Symmetry codes: (i) *x*, *y*−1, *z*; (ii) *x*+1, *y*, *z*; (iii) *x*, *y*+1, *z*.
